# Keratin-associated epidermolysis bullosa simplex: phenotypes and challenges in clinical trials – a narrative review and systematic update

**DOI:** 10.1186/s13023-025-03822-0

**Published:** 2025-06-20

**Authors:** Verena Wally, Tobias Welponer, Hans-Peter Wiesinger, Anja Diem, Konstantin Thiel, Martin Geroldinger, Georg Zimmermann, Julia I. Hummel, Sonja Dorfer, Josefina Piñón Hofbauer, Johann W. Bauer, Martin Laimer

**Affiliations:** 1 Research Program for Molecular Therapy of Genodermatoses, Department of Dermatology and Allergology, EB House Austria, University Hospital of the Paracelsus Medical University, Salzburg, 5020 Austria; 2 Department of Dermatology and Allergology, University Hospital of the Paracelsus Medical University Salzburg, Salzburg, 5020 Austria; 3 Master Programme Public Health, Center for Public Health and Healthcare Research, Paracelsus Medical University, Salzburg, 5020 Austria; 4 Institute of Nursing Science and Practice, Center for Public Health and Healthcare Research, Paracelsus Medical University, Salzburg, Austria; 5 Institute of General Practice, Family Medicine and Preventive Medicine, Paracelsus Medical University, Salzburg, Austria; 6 Department of Artificial Intelligence and Human Interfaces, Faculty of Digital and Analytical Sciences, Paris Lodron University, Salzburg, Austria; 7 Research Programme Biomedical Data Science, Paracelsus Medical University, Salzburg, Austria; 8 IDA Lab Salzburg, Team Biostatistics and Big Medical Data, Paracelsus Medical University, Salzburg, Austria; 9 Department of Biosciences and Medical Biology, University of Salzburg, Salzburg, 5020 Austria

**Keywords:** Epidermolysis bullosa simplex, Blisters, Clinical trials, Case reports

## Abstract

**Introduction:**

Clinical research on innovative therapies for the rare genodermatosis epidermolysis bullosa (EB) faces significant challenges, including small sample sizes, disease heterogeneity with intra- and inter-individual variability, limited understanding of pathogenic mechanisms and natural disease course, as well as the lack of patient-centred core outcomes. Moreover, existing tools and techniques to assess disease activity and dynamics are heterogeneous, inconsistent, and may fail to consider or inaccurately emphasize particularities of individual patients and distinct EB subtypes.

**Methods:**

In order to exemplify the differences between keratin-associated subtypes of EB simplex (k-EBS), we summarized respective clinical characteristics in a narrative way. In addition, we performed a systematic review of the literature published over the last 5 years, with the aim to give an overview on outcomes and their assessments used in these patient populations.

**Results:**

This review summarises the methodological scope, strengths and limitations of outcome assessments in clinical trials for the k-EBS, a group of inherited skin fragility diseases characterised by their distinct phenotype of epidermal blistering.

**Conclusions:**

By presenting an overview of the clinical spectrum of k-EBS, we identified key gaps in current assessment methodologies and propose alternative approaches to optimise the evaluation of skin blistering, with the aim of enhancing the accuracy, reliability, and patient-relevance of clinical outcomes.

**Supplementary Information:**

The online version contains supplementary material available at 10.1186/s13023-025-03822-0.

## Background

Epidermolysis bullosa (EB) is a major group of genetic disorders characterised by skin fragility. Within this group, EB simplex (EBS) is generally considered a mild subtype, although phenotypic differences across distinct EBS-subtypes are considerable [1, 2]. In recent decades, significant progress in the understanding of pathogenic mechanisms underlying EBS has facilitated major advancements in clinical research and approaches for translational therapy [3, 4]. However, clinical testing has revealed a plethora of challenges associated with drug development in EB, primarily due to the rarity of the disease, which results in small sample sizes and considerable heterogeneity within patient cohorts. Overcoming these obstacles requires careful consideration during the planning and execution of clinical research including an optimal study design, a clear definition of outcomes, and the selection of appropriate outcome measurement instruments.

In this review, we highlight the differences between patients suffering from different forms of EBS caused by keratin mutations (k-EBS), and summarise recent clinical research efforts on this subtype. We found that only a minor percentage of trials and case studies in the EB field specifically target EBS, which apply a wide variety of heterogeneous outcomes and measurement instruments. In addition, we identified subtype-specific peculiarities that complicate the conduct of large-scale clinical trials and propose strategies to address these methodological limitations.

## Main text

### Clinical characteristics of epidermolysis bullosa simplex

Epidermolysis bullosa simplex (EBS) is the most common type of the rare genodermatosis epidermolysis bullosa (EB), accounting for at least 70% of all EB cases [5]. EBS results from mutations in one of at least seven distinct genes involved in maintaining the structural and functional integrity of the epidermis [1]. The term keratin-associated EBS (k-EBS) refers to those EBS variants that are caused by mutations in either keratin 5 (*KRT5)* or *KRT14* [1]. This group includes the majority of EBS cases and encompasses localised (loc-EBS, previously Weber-Cockayne), intermediate (intermed-EBS, previously EBS-generalised intermediate, EBS Köbner), and severe EBS subtypes (sev-EBS, previously EBS-generalised severe, EBS Dowling-Meara). Clinical hallmarks include trauma- or friction-induced superficial intra-epidermal blisters, erosions and crusts (Table 1) [1, 6, 7].

**Table 1:** Clinical hallmarks of the major k-EBS subtypes [1]

	Localised EBS	Intermediate EBS	Severe EBS
**Blistering**	Single or grouped blisters	Single or grouped blisters	Spontaneous, typically herpetiform (arciform)
**Skin involvement**	Mainly restricted to mechanically exposed acral sites (hands, feet); onset at birth or in early infancy or adulthood; EB nevi	Generalised, but less severe; onset at birth or infancy; predilection for extremities and over bony prominences; EB nevi	Generalised, extensive; onset at birth; nail involvement (thickened, dystrophic); blistering tends to decrease and become more localised; EB nevi
**Other characteristics**	Inflammatory erythema; palmoplantar hyperkeratosis	Inflammatory erythema; palmoplantar hyperkeratosis	Inflammatory plaques; confluent palmoplantar hyperkeratosis
**Extracutaneous manifestations**	Rare	Rare	Oral mucosa; gastro-oesophageal reflux; growth retardation; perinatal complications (infection, malnutrition, respiratory failure)
**Pain**,** itch**, QoL*: Patient reported clinical characteristics [5, 10]	Pain: 66.7% Itch: 35.9% QoL13.4 ± 6.8	Pain 84.2%. Itch: 73.7% QoL: 15.8 ± 6.2	

While blistering is the leading feature of EBS, there is a pronounced phenotypic variability among EBS-subtypes with regard to healing course, extent of inflammation and skin involvement as well as extracutaneous manifestations. *QoL scores. Very mild (0–4), mild (5–9), moderate (10–19), severe (20–34), and very severe (35–51)

#### Localised epidermolysis bullosa simplex

**Fig. 1:**
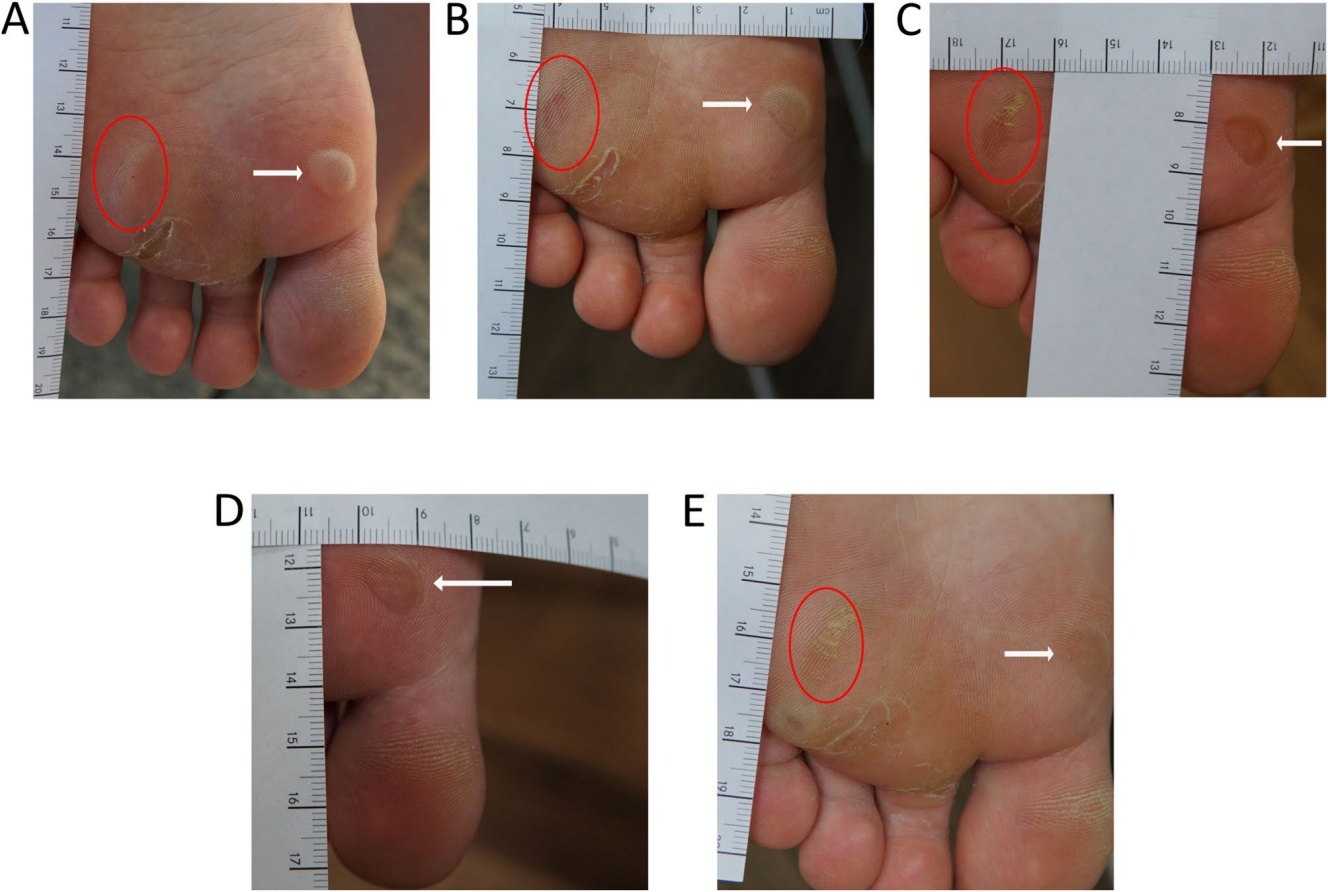
Localised EBS. 22-year old female patient with localised EBS (KRT14; c.1162 C > T; p.R388C). Healing course of an acute blister (white arrow) during a nine-day observation period. (**A**) Day 1, (**B**) day 3, (**C**) day 5, (**D**) day 7, (**E**) day 9. Red circle indicates a healing blister that pre-existed already at the beginning of documentation in the area of interest

Localised EBS (loc-EBS) is the most common form of EB, with a reported prevalence of 3.94 and 11.40 individuals per 1 million population in the United States and United Kingdom, respectively [8, 9]. It typically presents at birth or early infancy, but may not manifest until adulthood in some cases. Patients develop tense blisters upon mechanical stress. As the mildest variant, lesions in loc-EBS are largely limited to acral sites of the hands and feet. While the blister roof usually remains intact until healing is complete, it occasionally perforates, either spontaneously or subsequent to mechanical stress, resulting in erosions. Open wounds typically heal within several days without scarring or milia formation. Rarely, wound healing may be complicated by painful hyperkeratosis (Fig. 1) [10]. Nail dystrophy is uncommon and generally mild when present. In contrast, palmoplantar hyperhidrosis is frequent in patients. Blistering or ulceration of the oral mucosa, which can be triggered by bottle-feeding in infancy, typically resolves with age. Hair and teeth are usually unaffected.

#### Intermediate epidermolysis bullosa simplex

**Fig. 2:**
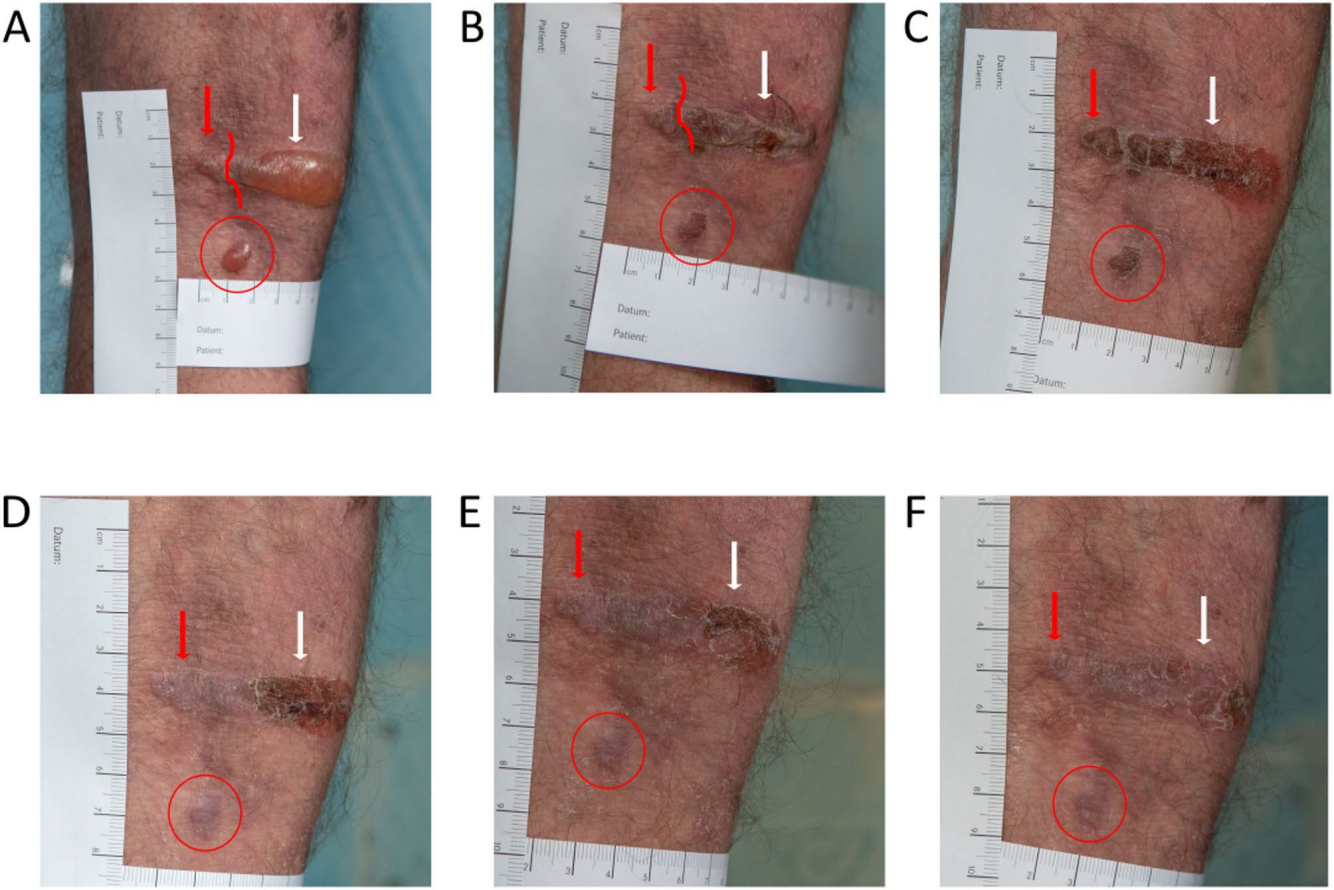
Intermediate EBS. Confluence of two adjacent, previously separated blisters in the popliteal fossa (red and white arrow, fusion zone indicated in red) in a 28-year old patient with KRT14 mutated intermed-EBS (c.744delC/insAG; p.Y248*). (**A**) The confluent lesion from day 1 had healed gradually from the medial to the lateral portion with crusting until (**F**) day eight. (**B**) Day 3, (**C**) day 5, (**D**) day 6, (**E**) day 7. An additional pre-existing blister in the proximity (red circle) shows complete healing at day six

In patients with intermed-EBS, blistering typically begins at birth or in early infancy. The condition is generalised (although blisters tend to be grouped, with a coalescing distribution), most prominently affecting the skin on the hands, feet, extremities, and over bony prominences (Fig. 2). Single blisters generally heal within a few days, often with post-inflammatory hyperpigmentation. Skin atrophy and milia may occur, though less frequently than in severe subtypes of EBS. While hair and teeth typically appear normal, nails may become thickened or dystrophic.

#### Severe epidermolysis Bullosa simplex

**Fig. 3:**
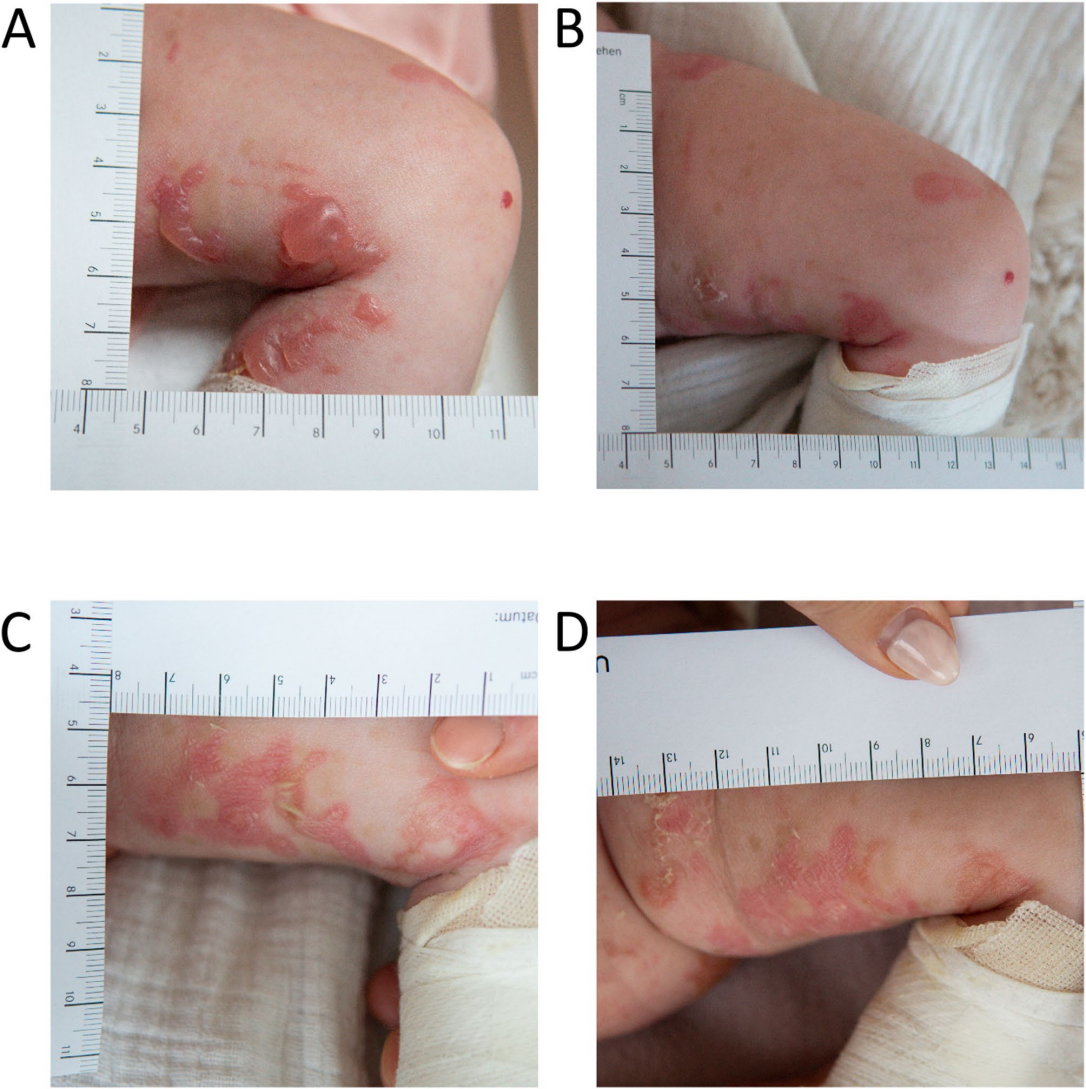
Severe EBS. Irregularly shaped and confluent blisters on the lower extremity of a seven-month old infant with severe EBS and mutation in the KRT14 gene (c.356T > C; p.M119T). Intact blister roof, irregular and confluent shape as well as inflammation impede unequivocal determination of blister borders and size of erosive areas. (**A**) Day 1, (**B**) day 2, (**C**) day 4, (**D**) day 5

Sev-EBS presents at birth with widespread and often spontaneous formation of large blisters and erosions (Fig. 3). Blisters tend to be grouped and coalesce, resulting in irregularly shaped lesions (herpetiform or arciform). Erosions and inflammatory erythema are prominent features.

Congenital absence of skin with ulcerations on the extremities as well as oral involvement may occur during infancy. While disease activity is severe and debilitating in the first years of life, with an increased risk of life-threatening complications (e.g., respiratory failure, malnutrition, electrolyte imbalance, fluid loss, infection, and sepsis), symptoms tend to improve with age. Over time, blisters become smaller and, particularly when healing, display a characteristic herpetiform conformation. During infancy, hyperkeratosis of the palms and soles often develops and may progress to painful, confluent keratoderma, impairing motor development (e.g., ability to walk) and quality of life (QoL). While erosions usually heal without scarring, post-inflammatory hypo- or hyperpigmentation are common. Milia and skin atrophy mainly occur in infancy. Notably, besides friction and mechanical trauma, factors such as dysbiosis, hot climate, high temperatures and sweating, can exacerbate disease expressivity by affecting number, distribution, conformation, healing course and localisation of blisters.

Additional clinical features include nail dystrophy and nail shedding. Extracutaneous manifestations can be severe (e.g., laryngeal stenosis) and may result in fatal complications [1, 11].

### Pathogenesis of keratin-associated EBS

K-EBS is primarily caused by autosomal dominant missense mutations in genes encoding either keratin 5 (*KRT5)* or *KRT14* [1]. These keratins are expressed in basal keratinocytes and form the intermediate filament (IF) network, which is essential for mechanical strength and resilience of the epidermis [2]. Approximately 30% of *KRT5/14* mutations arise *de novo*, which is considerably high. Although rare, k-EBS can also follow an autosomal recessive inheritance pattern, particularly in *KRT14* variants [1, 12–17]. Moreover, semi-dominant inheritance, variable penetrance, and digenic occurrence of pathogenic variants in both *KRT5* and *KRT14* have also been reported [18–20]. In some cases, large intragenic *KRT5* or *KRT14* mutations have been implicated [21].

Overall, the type (homozygosity versus heterozygosity), number (monogenic or digenic inheritance), and location of mutations within the respective keratin genes or gene segments, contribute to the considerable genetic heterogeneity of k-EBS [1]. These factors influence the extent of quantitative (absence, reduction, increase) and qualitative (gradual loss or gain of function) alterations in protein expression, giving rise to the variable phenotypic spectrum of the disease.

The type and localisation of *KRT*-mutations are the major determinants of disease severity. Generally, mutations in keratins disrupt protein heterodimerisation, affecting its proper integration into hemidesmosomes and desmosomes and destabilizing the intermediate filament network. The severity of this disruption is largely dependent on the protein domain that is affected by the respective mutation, with molecular aberrations in highly conserved regions associated with the more severe EBS subtypes. Consistently, mutational hotspots in the highly conserved helix-formation domains are associated with sev-EBS. Mutations in these domains lead to a collapse of the IF network upon trauma, resulting in the formation of dense keratin aggregates that trigger inflammatory stress signatures with pro-inflammatory cytokines IL-1ß and IL-17 as key mediators [22–24]. Conversely, mutations in less conserved areas, such as the head or tail domains of the protein, result in impaired but partially preserved filament formation and function. Such variants are associated with a milder (localised) phenotype [2]. Particularly rare subtypes of k-EBS are EBS with mottled pigmentation, which is mostly caused by *KRT5* mutations, and EBS with migratory circinate erythema [1, 25, 26].

### Current care standards and therapeutic prospects

#### Current standard of care

Despite its physically and psychologically debilitating symptoms accounting for a substantial disease burden in patients, no causal therapy specifically developed for k-EBS has been approved. Current treatment approaches focus on alleviating the most burdensome symptoms, i.e., blistering, pain, and itch [5]. Supportive care focuses on protection and avoidance of triggering factors, wound care to improve healing of blisters and erosions, control of microbial burden and infection, alleviation of pain and itch, nutritional supplementation, psychological support, occupational therapy, and monitoring and management of complications (e.g., anaemia, skin cancer) [27, 28].

#### Therapeutic prospects

While EBS represents the largest cohort within the EB population by far (reported prevalence: 6 and 17 / 1 million population, respectively) [8, 9], it remains underrepresented in clinical research. A recent scoping review revealed that only 21 out of 207 (10.1%) clinical studies conducted in the EB field between 1991 and 2021 included patients with EBS. Moreover, while the overall number of clinical trials in EB has steadily increased over time, the number of trials for EBS has remained relatively stable [29]. A systematic review of the literature [additional file 1] and clinical trial databases [additional file 2] of the last 5 years identified only five case reports on EBS (Table 2) and seven randomized controlled trials (RCTs, Table 3) that have been published or registered. Unlike trials for other EB-types, which primarily focus on wound healing, trials in EBS have instead prioritised different outcomes - such as body surface area (BSA) affected, investigator global assessment (IGA), and itch and/or pain scores - which reflect the typical clinical features of this specific EB-subtype (Table 4).

**Table 2:** Case studies investigating drugs for EBS published since 2020

Treatment	Observations	No of patients	Reference
Cisplatin	Despite being given for the treatment of nasopharyngeal carcinoma in an EBS patient, there was a total clearance of existing blisters with no recurrence during time of observation.	1	[30]
1% Sirolimus	Significantly reduced blistering and keratoderma, improved ambulation and decrease of plantar pain.	2	[31]
Dupilumab	Reduction in blistering, erosions and IgE levels, improvement in skin fragility.	1	[32]
Botulinum toxin	Decrease in blister formation lasting 6 months to one year, improvement of quality of life.	2	[33]
Dapsone	Notable improvement in disease activity, with a reduction in the EBDASI-activity score, sustained at the 7-month follow up period, subjective palmar improvement in his palmar keratoderma, with increasing palmar desquamation	1	[34]

Case studies were identified by systematically searching PubMed and Google Scholar databases [additional file 1]

**Table 3:** Randomized-controlled clinical trials registered between 2021 and 2024 for EBS

Treatment	Subtypes	Number of patients (EBS/total)	Primary Outcome Measure	Secondary Outcome Measure	Reference, status
TolaSure (BM-3103)	Severe EBS	6/6	Incidence of Treatment-Emergent Adverse Events (TEAEs).	Investigator Global Assessment (IGA).	NCT05062070, completed, no results posted.
				Keratin aggregate counts and cell morphology assessment.	
				Suction blister time.	
				Patient’s-reported overall impression score of treatment areas.	
				Patient’s-reported pain score of treatment areas.	
				Patient’s-reported itch score of treatment areas.	
				Proportion of keratinocytes containing autophagosomes.	
				Cytokine content in suction blister fluid.	
Diacerein (AC-203)	EBS	54/54	Proportion of subjects who achieved ≥ 60% reduction in Body Surface Area (BSA) of EBS lesions within assessment area.	Proportion of subjects who achieved success on the Investigator’s Global Assessment (IGA)	NCT03154333, terminated. Study was discontinued upon interim analysis [38].
Allantoin (SD-101)	EBS, JEB, RDEB	18/169	Time to complete target wound closure within 3 months.	Percentage of participants experiencing complete closure of their target wound at month 1 and month 2 visits	NCT02384460, completed. No significant differences between SD-101 6% and vehicle were observed for the primary efficacy endpoint [54].
			Percentage of participants experiencing complete closure of their target wound within 3 months.	Change from baseline in Body Surface Area Index (BSAI) of lesional skin at month 3 visit.	
				Change from baseline in BSAI of total body wound burden at month 3 visit.	
				Change from baseline in itching score at day 7.	
				Change from baseline in pain score at day 7.	
Cannabinol (INM-755)	All types of EB	unknown/19	Change from baseline in non-wound itch by Visual Analogue Scale (VAS).		NCT04908215, completed. No results available.
			Change from baseline in wound surface area.		
			Change from baseline in procedural wound pain by VAS.		
			Change from baseline in background wound pain by VAS.		
			Change from baseline in wound itch by VAS.		
Diacerein (AC-203)	Generalized EBS	80 (planned)	Proportion of patients achieving treatment success on the IGA of the treatment area, in which treatment success is defined as a score of 0 or 1 with at least a 2-point reduction.	Change in % BSA of EBS lesions in the treatment area.	NCT06073132, recruiting
				Change in pain intensity score.	
				Change in pruritus intensity score.	
				Change in EBDASI score (skin activity).	
				Change in the QOLEB score.	
Botulinic Toxin	Localised EBS	25 (planned)	Global clinical improvement on each foot assessed by a blinded centralized independent reviewer using photographs, at M3 vs. baseline: IGA score (Improvement Global Assessment) assessed for each foot.	Global clinical improvement on each foot assessed by a blinded centralized independent reviewer using photographs: IGA score (Improvement Global Assessment) for each foot.	NCT03453632, recruiting.
				Global clinical improvement on each foot assessed by the investigator: IGA score (Improvement Global Assessment) assessed for each foot.	
				Efficacy assessment concerning the number of plantar lesions clinically observed by the investigator.	
				Efficacy assessment on each foot of the affected plantar skin surface (blisters, erosions, erythematous and oedematous areas, crusts).	
				Efficacy assessment by the patient himself, for each foot.	
				Plantar pain assessment by the patient himself, for each foot.	
				Immediate tolerance during injection.	
				Mid-term and long-term tolerance.	
Sirolimus	EBS	8/8	Foot health status questionnaire, foot function domain score.	Average steps per day assessed by FitBit^®^ / pedometer.	NCT02960997, completed. Results published on clinicaltrials.gov. No significant differences in primary endpoint.
			Foot health status questionnaire, physical activity domain score.	Child dermatological quality of life questionnaire score.	
			Trough concentration of Sirolimus.	Epidermolysis Bullosa Disease Activity and Scarring Index (EBDASI) disease severity scale score.	
				5-D pruritus scale score.	
				Plantar defect size using 3D photography.	
				Foot plantar pressure measurements.	
				Change in mTOR pathway inhibition.	

Currently registered, recruiting or ongoing trials for EBS only or including patients with EBS were identified by screening FDA- (www.clinicaltrials.gov) and EMA-hosted registries (https://www.clinicaltrialsregister.eu) [additional file 2]

Of note, the limited evidence on efficacy and safety of investigational drugs for EBS relies mostly on case reports and a few clinical trials involving small cohort sizes and typically lacking a control arm [29, 35]. This historical shortcoming may be an indirect consequence of the overall milder phenotypic expressivity of EBS, which has led to a greater research focus on the more severe types of EB, particularly dystrophic EB (DEB). Additionally, methodological bottlenecks render clinical research activities for EBS challenging.

**Table 4:** Summary of RCT trials identified through the clinicaltrials.gov registry and literature search

**Population (number of studies)**	EBS only EBS + other EB types	*n* = 5 *n* = 2
**Number of participants**		*n* = 361 (min. 6, max. 169)
Number of participants with EBS*		n (EBS) = 166 / 317 (min. 6, max. 80)
**Application**	Topical treatment: Injections:	*n* = 6 *n* = 1
BSA**		4/7
**IGA**		4/7
**Blisters**		2/7
**Questionnaires (non EB-specific)**		1/7
**EB-specific scoring systems (EBDASI**,** QOLEB)**		2/7
**Itch**		5/7
**Pain**		5/7
**Wound healing**		2/7

*Studies excluded: recruiting (*n* = 2) or unknown number of EBS participants (*n* = 1). ** Whole body or restricted to distinct parts of the body (e.g., feet)

### Methodological bottlenecks

Given the extensive phenotypic scope of EB, a thorough understanding of the clinical characteristics of distinct EB types is essential for defining accurate trial endpoints and designs. “Wound healing”, with particular reference to longer-standing (chronic, refractory) wounds, has been the most commonly assessed outcome domain (with domain referring to a group of similar outcomes) [29] in EB clinical research. However, EBS patients rarely develop wounds nor exhibit wound healing deficits. Instead, affected individuals typically experience a highly dynamic course of blister formation and healing, which varies both across patients and within the same individual over time. Consequently, studies with EBS patients have primarily assessed reduction in blister formation as the primary endpoint (66.7%) and have further reported on measures to monitor blister development and healing time (9.6%). In addition to the limited volume of clinical trial data on k-EBS, there is a considerable heterogeneity in the outcome domains and measurement instruments applied in these studies. A total of 21 outcome domains have been reported [29], with studies published or registered since 2020 introducing additional endpoints to this list (Tables 2 and 3).

#### Evaluation of skin blistering

Since skin blistering is the predominant and most patient-relevant symptom in k-EBS [5], various approaches to evaluate the impact of a therapeutic intervention on blister numbers have been described.

The selection of a distinct measurement instrument depends, for instance, on the precise definition of the outcome to be evaluated in a study (e.g., “reduction of total number of blisters”, “reduction of time to healing”, “prevention of new blister development”). In general, blister numbers were commonly used to assess changes from baseline. Some studies additionally monitored blister evolution over a certain time period to support the validity of blister counting against the background of a highly dynamic lesional course in EBS characterised by phases of evolution, healing and recurrence. Blister counting was assessed in real time or based on photos (e.g., Wally et al., JAAD) [36]. Overall, the authors concluded that accurately monitoring blister numbers was highly challenging due to the specific healing course of EB blisters, which typically involved a large blister evolving into a cluster of smaller blisters during the healing process.

#### Investigator’s global assessment (IGA)

IGA scales were developed to evaluate the average overall severity of EBS lesions in clinical trials [37]. They typically comprise a set of relevant features that reflect the severity of EBS lesions, such as the presence or absence of blisters, erosions, crusting and erythema, and blister size. By combining these features into a single metric, IGA scales provide an assessment of EBS-lesional skin, independent of the particular healing course of EBS blisters.

These instruments have been used in studies testing various treatments, such as diacerein (DELIVERS study, NCT03154333 [38],, botulinum toxin (NCT03453632), and the anti-bacterial compound BM-3103 (4-Hydroxy-4’-methyoxytolan, NCT05062070), which also involve patients with k-EBS. A key methodological challenge with IGA-scales is the inter-rater variability in assessments, which can be addressed by providing clear, unambiguous/unequivocal definitions of the items included in the scale.

#### Body surface area (BSA) assessments

Overall BSA involvement was evaluated in four out of seven registered RCT trials for k-EBS conducted between 2020 and 2024 (Table 4). The body areas assessed varied, with some studies restricting evaluation to certain sites like the hands and feet, while others considered the entire body surface. Lesional BSA was estimated either by using the palmar method (where the size of a patient’s palm corresponds to 1% BSA in children and 0.5% in adults) (e.g., NCT03154333) [38], or by photography and subsequent calculation. Photography-based assessments, however, were performed only for smaller body areas (e.g., for botulinic toxin, NCT03453632).

A major issue with BSA assessments is standardisation, as large inter-rater variability can occur [39–41]. In this context, studies in patients with psoriasis found that while intra-rater variability was generally low, inter-rater variability was particularly high when using the “rule-of-9” or “palmar” method. Consequently, while BSA assessment may be well suited for longitudinal measurements by the same physician, caution is advised when different raters assess the extent of lesional involvement on the body surface.

Similarly, photography-based assessment of lesional BSA may be impaired by inadequate inter- and intra-patient standardisation, such as insufficient resolution of images and lack of sensitivity in capturing 3D-concavities [42]. Notably, previous experience has shown that slight angle deviations of only a few degrees when taking serial photos can result in significant differences in 2D skin surface area evaluation (our unpublished data). Body concavities, such as armpits, are particularly prone to blistering in EBS patients, but are difficult to capture with most available tools. Thus, when selecting outcomes, the pros and cons of the different available technologies must be carefully considered.

Innovative digital imaging systems with AI-based automation of pattern analysis hold some promise for more reliable, rater-independent assessment of affected BSA. While significant technological advances have been made in this field, these techniques, and particularly total body and/or three-dimensional scanning, may be cost-intensive and require adequate infrastructure. Digital photography can provide various types of information (e.g., lesion localisation, size measurements). Moreover, the integration and central analysis of remotely acquired (e.g., at home) images may also significantly reduce trial and travel burden of EB patients participating in clinical studies [43].

#### Disease activity scores

Various scoring systems have been developed to comprehensively assess disease severity and activity during treatment. Ideally, these instruments provide a standardised and intuitive tool applicable for all EB-subtypes and ages. Scoring indices, such as the Birmingham EB Severity (BEBS) Score [44], the EB Disease Activity and Scarring Index (EBDASI) [45] and the Instrument for Scoring Clinical Outcomes of Research for EB (iscorEB) [46, 47], have all been analysed for their reliability and validity. Notably, these instruments demonstrate crucial differences in.

- their ability to differentiate between disease activity and (inert, chronic) damage,
- their assessment of the extent of extracutaneous disease involvement,
- their approach to defining skin involvement, and.
- the impact of secondary manifestations on the final score.

While the applicability of these scores to all EB types is beneficial, their utility to assess treatment success in k-EBS is limited. This is because only a subset of the assessment criteria is applicable to the phenotype of this distinct patient cohort. Consequently, the sensitivity to measure intervention-induced changes in skin blistering is diminished, as treatment effects may be outweighed by the impact of criteria for which EBS patients inherently receive zero points, such as the presence of chronic wounds or carcinomas and the involvement of other organs. To address this issue, one strategy is to use only those subsections of the scoring tool that are relevant to k-EBS. However, this approach currently lacks independent validation.

#### Other outcome measures

Additional outcomes that have been measured in clinical studies for EBS include skin resilience, cancer formation, lesional infection rates, various safety aspects, as well as patient-reported outcomes such as pain, pruritus, and quality of life. This diversity in outcomes measured has made it challenging to compare results and draw meaningful conclusions about treatment efficacy across the various studies. To enable better standardisation and comparison, a core outcome set (COS) for EBS, which identifies the most critical outcome domains (“what” to measure) and the corresponding outcome measurement instruments (“how” to measure), is currently being developed by an international group of EB stakeholders [29, 48]. By reducing variability and harmonising reporting, the use and consistent reporting of such COSs should facilitate accurate comparison and pooling of data, ultimately expediting therapy development [48].

### Challenges in clinical research

The definition of research questions, trial design, and the selection of outcomes, along with the use of appropriate measurement instruments that are (a) representative of the EB subtype and its associated (distinctive) symptoms and (b) accepted by all trial stakeholders, including patients and regulatory authorities, is essential for validating the efficacy of a given treatment and preventing trial failures due to methodological shortcomings. The challenges associated with these aspects in the context of EBS as a rare disease are summarised in Table 5 and include: difficulties in defining key milestones and suitable endpoints, determining an appropriate study length, estimating accurate sample and effect sizes, developing clinical rating scales to quantify disease activity/progression, establishing the types and timing of validated outcome measures. Notably, most of these aspects are intertwined and impact each other. For example, sample size planning influences patient recruitment, as does the development of trial protocols with an associated burden that is manageable for patients.

**Table 5:** Challenges associated with clinical research in the rare disease setting of EBS

	Description	Challenges
Trial design	Randomised-controlled trials (RCT) provide the highest level of formal confidence. Innovative trial designs may be necessary for EBS studies [51].	RCT trials are usually expensive with a limited commercial interest, time-consuming and require a high number of patients, given the inclusion of at least one control group. Data quality and quantity necessary to provide reliable information on the efficacy and safety of a drug needs to be ensured. Definition of key milestones and suitable endpoints. Selection of the appropriate study length. Timing of validated outcome measures.
Sample size planning	Sample size planning (SSP), which reflects the requirement of certain patient numbers to achieve a high probability of detecting a statistically significant effect (i.e., statistical power) with regard to the primary endpoint, should be routinely done for clinical trials, but also for observational studies (for the latter, a different framework that is focusing on coverage probabilities for confidence intervals is usually preferable), to provide statistical power and accuracy, but also due to ethical and economic reasons.	High drop-out rates are common to EB trials. Lack of data as a solid basis for power analysis / sample size calculation.
Patient recruitment	For EBS, being a rare disease, low numbers of patients are available or accessible.	Some patients are in a particularly poor condition. Patients are typically geographically dispersed. Internal and external competition for eligible probands. Recruitment failures include reasons like missing diagnosis, lack of patient awareness and literacy, as well as patient reluctance to participate due to demanding protocols. Low sample sizes might lead to a considerable decrease of statistical power.
Phenotypic variability	Particularly heterogeneity, i.e.,* the* variability of phenotypic expression at various levels (inter-patient/-subtype, intra-patient) can negatively impact assessment trial outcomes. Inclusion criteria, for instance, define the minimal number of blisters / lesional burden at baseline.	Selection of clinical rating scales to quantify disease activity/progression/variation and types. Account for considerable heterogeneity in the statistical analysis.

## Conclusion

The considerable genetic and phenotypic heterogeneity of EB simplex subtypes, combined with their highly dynamic natural course, poses a significant challenge to clinical research and the accurate validation of innovative therapeutic treatments. Disease activity and outcomes in EBS can be modified by environmental (e.g., seasonal impacts), behavioural (e.g., activity) and molecular (e.g., activation of inflammatory pathways) factors. These complexities exemplify the broader challenges faced in EB drug development.

The importance of accurate patient stratification and targeting, even within the k-EBS cohort, is underscored by a phase II/III clinical trial that was terminated at interim-analysis due to the absence of a statistically significant effect of the drug over placebo. Remarkably, post-hoc analysis attributed this failure to the inclusion of patients with differing k-EBS subtypes, which resulted in varying susceptibilities to the therapeutic intervention [38]. In another trial, EBS patients were excluded, following an amendment, based on their distinct phenotypic presentation which was deemed likely to considerably impact study outcomes. This exclusion was justified as EBS typically displays a rather mild course compared to other EB forms, with shorter wound healing cycles, spontaneously-healing size-limited lesions, and the absence of chronic wounds lasting more than 3 weeks. Inclusion of EBS patients would have disproportionately favoured the control arm of the study, illustrating the importance of understanding EB and even EBS subtype-specific particularities, especially in the context of clinical testing [4].

Although having the highest prevalence among all EB subtypes, EBS patients are under-represented in either published or registered clinical studies, when comparing to the overall trial landscape in EB [29]. This may reflect the perception that, since EBS exhibits an overall milder phenotypic expressivity compared to severe EB types, it is associated with a lower medical need. Furthermore, while in most EB-types accumulation of damage and loss of regenerative capacity result in a chronic and/or progressive course, EBS patients often experience symptomatic amelioration over time. As such, the optimal time window for innovative treatments may be limited to younger ages in k-EBS. However, this primary target group typically falls into an age range that is commonly excluded from (pilot) studies due to regulatory safety aspects. In older patients, the burden of trial participation may outweigh the potential clinical benefits, thereby negatively impacting patient enrolment. All these factors may additionally discourage financial and pharmaceutical industry investment.

Other methodological bottlenecks also hinder clinical trials for EBS (Table 6). A major limitation is the lack of well-established outcomes for EBS, particularly for monitoring of blistering. This is reflected in the variety and inconsistent use of heterogeneous skin-related outcomes in clinical trials for EBS, as identified by the systematic review by Korte et al. and further substantiated by our findings [29]. The development of core outcomes that are suitable, patient-centred, and sensitive to therapeutic intervention within a reasonable timeframe is critical. Equally important is the availability of objective measurement tools to assess these outcomes. Capturing all relevant aspects/dimensions/qualities of the interventional impact, including those deemed necessary by regulatory authorities is essential. In recent years, much effort has been placed on developing instruments to accurately measure parameters such as body surface area and blister area. While validated tools are already available/accessible (e.g., visual analogue scales, questionnaires addressing quality of life), the current tools do not sufficiently address the unique characteristics of the distinct EB subtypes (e.g., objective measurement of blistering, total body assessment). Technological advancements, such as total body stereographic 3D-digital photography and the development of digital image analysis to objectively assess disease activity and the impact of therapeutic interventions, will represent key steps forward in the validation of innovative drugs in skin-related outcomes.

**Table 6:** Proposals addressing challenges in clinical trials for EBS

Challenge	Approaches
Trial design	Cross-over designs and intra-patient controlled trials are attempts to reduce sample size and to minimise confounding factors. However, these approaches create new challenges. For instance, stratification into subgroups will most likely increase the number of subjects that need to be enrolled in order to increase the probability of reaching statistical significance. Moreover, carry-over effects may affect the outcomes. Cross-over trial designs further prolong the overall duration of a clinical trial which in turn may reduce patients’ commitment and compliance. Importantly, in the rare disease field, innovative designs have been successfully used in pivotal trials and were accepted by authorities [49, 50, 52, 53].
Sample size planning (SSP)	More flexible study design and planning concepts (e.g., adaptive designs with interim analyses), more robust sample size planning approaches that allow for correcting initial misspecifications or uncertainties, conducting pilot studies or exploiting historical data, in order to provide reasonable estimates, for example, of the variability of the selected outcome measure.
Patient recruitment	Minimising the number of visits needed. Inclusion of a visiting study nurse [43]. Applying analysis methodologies that are particularly tailored for small samples.
Phenotypic variability	Above mentioned cross-over designs or simultaneous intra-patient control are both additional options to reduce the effects of potential confounding variables (e.g., season, age) [49]. Inter-subtype variability can be addressed by separating distinct EBS-subtypes through inclusion/exclusion criteria or by stratification of patients.
Lack of blister-related outcomes and validated measurement instruments.	Need for the development of EBS-representative, valid and expert-consented outcomes, particularly for blister evaluation/monitoring, together with objective means of outcome measurement.

In summary, given the need for effective treatment options for all types of EB, including EBS-subtypes, it is essential to take into account various subtype-specific clinical, pathogenic, and molecular characteristics when designing clinical trials. Careful selection of the target population and the corresponding appropriate outcomes will help ensure the validity and reliability of clinical testing and assessment of a therapeutic effect. Innovative study concepts, as alternatives to the classical parallel-group clinical trial design (e.g., cross-over studies, intra-patient controls and stratification of patients), hold promise in addressing bottlenecks inherent to rare disease clinical research [49–53]. Moreover, collaborative and consortia efforts, including partnerships with regulatory agencies and industry, will be crucial to accelerating the development and market availability of promising treatments for the various EB subtypes.

## Electronic supplementary material

Below is the link to the electronic supplementary material.

Supplementary Material 1

Supplementary Material 2

## Data Availability

All data generated or analysed during this study are included in this published article.

## Abbreviations

EBS: Epidermolysis bullosa simplex
QoL: Quality of Life
IGA: Investigator’s Global Assessment
BSA: Body Surface Area
COS: Core Outcome Set
loc: Localised
sev: Severe
intermed: Intermediate
k-EBS: Keratin-associated epidermolysis bullosa simplex
EB: Epidermolysis bullosa
IF: Intermediate filament
KRT: Keratin
DEB: Dystrophic epidermolysis bullosa
BEBSS: Birmingham EB Severity Score
EBDASI: EB Disease Activity and Scarring Index
iscorEB: Instrument for Scoring Clinical Outcomes of Research for EB
