# Supplementary material for: Keratin-associated epidermolysis bullosa simplex: phenotypes and challenges in clinical trials – a narrative review and systematic update
Source: Orphanet J Rare Dis. 2025 Jun 20;20:313. doi: 10.1186/s13023-025-03822-0 (PMC12180272; doi:10.1186/s13023-025-03822-0)
Supplement: Supplementary file 1 — Supplementary Material 1 [file 13023_2025_3822_MOESM1_ESM.docx]

**Additional file 1: Search strategy and study selection in publication databases.**

Literature screening was done by two independent reviewers based on the below criteria, for import into the Rayyan software (Doha, Qatar [1]) [1].

| **Database** | **Search String and Filters** | **Results** |
| --- | --- | --- |
| PubMed | "Epidermolysis bullosa simplex" NOT "dystrohpic" NOT "junctional" NOT "carcinoma"  Filters applied: Abstract, Case Reports, Clinical Study, Clinical Trial, Clinical Trial, Phase I, Clinical Trial, Phase II, Clinical Trial, Phase III, Clinical Trial, Phase IV, Randomized Controlled Trial. Publication time: 2020 – 2024. | 27 |
| PubMed | “Epidermolysis bullosa simplex” AND “treatment”  Filters applied: Case Reports, Clinical Study, Clinical Trial, Clinical Trial, Phase I, Clinical Trial, Phase II, Clinical Trial, Phase III, Clinical Trial, Phase IV, Randomized Controlled Trial. Publication time: 2020 – 2024. | 11 |
| GoogleScholar | treatment "clinical trial" OR "clinical study" OR "case report" OR "case study" "epidermolysis bullosa simplex" -cancer -carcinoma -review | 54 |

**Inclusion criteria:**

- Skin-related outcomes.
- Publications: 2020 – 2024.
- Population: mixed populations when including EBS.

**Exclusion criteria:**

- Systematic review, scoping review, meta-analysis.
- Pre-clinical studies.
- Investigation of dressings.
- Population: Junctional EB, Dystrophic EB, patients with other diseases.
- Studies on incidence and prevalence.
- Qualitative studies.
- Conference abstract.

**Screening:**

Screening of titles, abstracts and full-text reports was performed by two independent reviewers (V.W., S.D.) using Rayyan (Doha, Qatar, [1]).

**PRISM:**


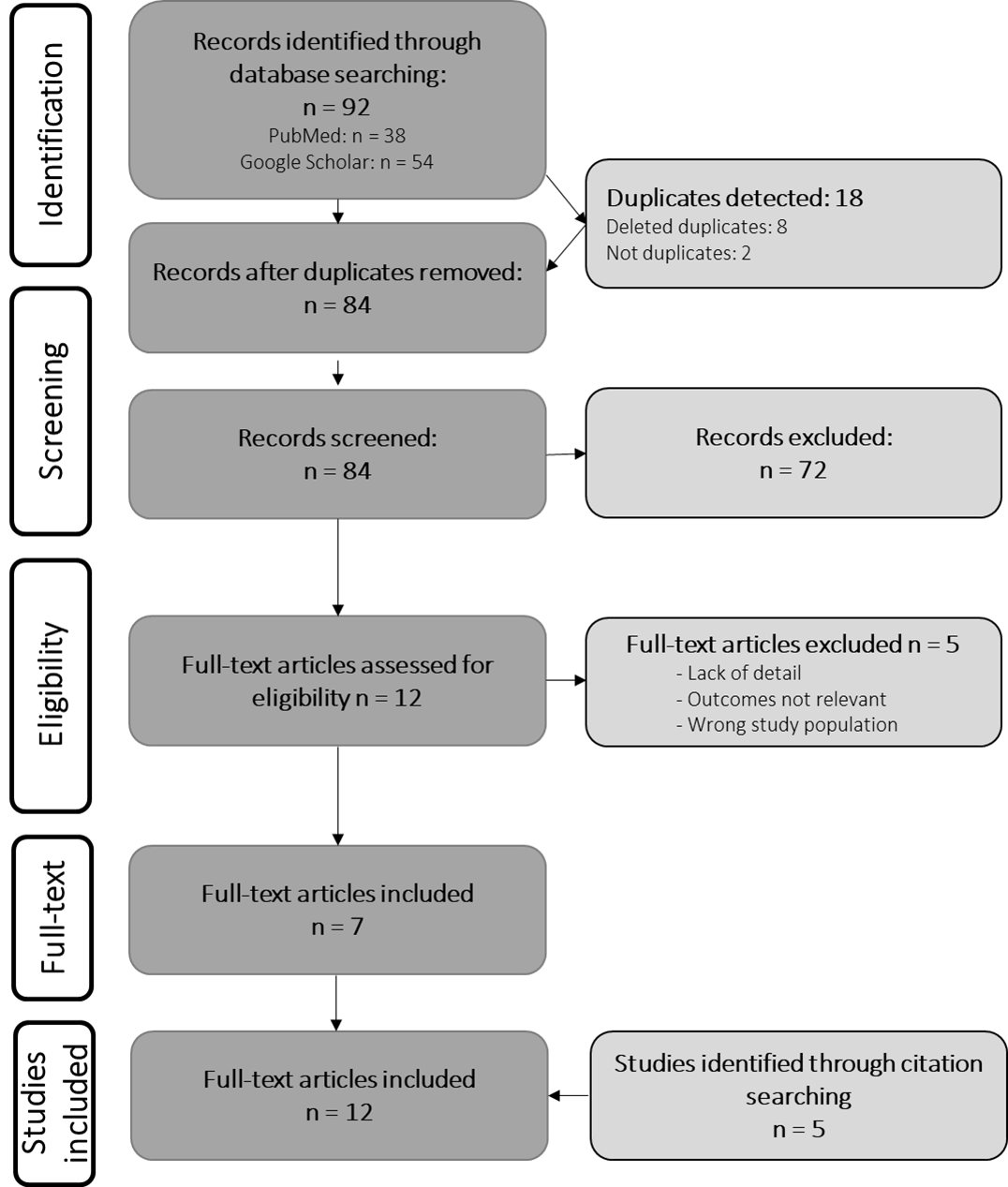


References:

1 Ouzzani M, Hammady H, Fedorowicz Z, Elmagarmid A. Rayyan-a web and mobile app for systematic reviews. *Syst Rev* 2016; 5: 210.
