# Supplementary material for: Keratin-associated epidermolysis bullosa simplex: phenotypes and challenges in clinical trials – a narrative review and systematic update
Source: Orphanet J Rare Dis. 2025 Jun 20;20:313. doi: 10.1186/s13023-025-03822-0 (PMC12180272; doi:10.1186/s13023-025-03822-0)
Supplement: Supplementary file 2 — Supplementary Material 2 [file 13023_2025_3822_MOESM2_ESM.docx]

**Additional file 2. Search strategy and study selection in clinical trial databases.**

For the identification of trials in clinical trial databases, currently registered, recruiting or ongoing trials for EBS were identified by screening FDA- (www.clinicaltrials.gov) and EMA-hosted registries (https://www.clinicaltrialsregister.eu). Screening was done by two independent reviewers. Data was extracted to an excel-file, with pre-defined criteria (*e.g*., sample size, type of study, type of intervention, outcomes, outcome measurement, etc.). From this file, information included in this review was extracted in a qualitative and/or quantitative manner.
